# Mosquito bite immunization with radiation-attenuated *Plasmodium falciparum* sporozoites: safety, tolerability, protective efficacy and humoral immunogenicity

**DOI:** 10.1186/s12936-016-1435-y

**Published:** 2016-07-22

**Authors:** Bradley W. Hickey, Joanne M. Lumsden, Sharina Reyes, Martha Sedegah, Michael R. Hollingdale, Daniel A. Freilich, Thomas C. Luke, Yupin Charoenvit, Lucy M. Goh, Mara P. Berzins, Lolita Bebris, John B. Sacci, Patricia De La Vega, Ruobing Wang, Harini Ganeshan, Esteban N. Abot, Daniel J. Carucci, Denise L. Doolan, Gary T. Brice, Anita Kumar, Joao Aguiar, Thomas B. Nutman, Susan F. Leitman, Stephen L. Hoffman, Judith E. Epstein, Thomas L. Richie

**Affiliations:** US Military Malaria Vaccine Program, Naval Medical Research Center, Silver Spring, MD USA; Henry M. Jackson Foundation for the Advancement of Military Medicine, Rockville, MD USA; Laboratory of Parasitic Diseases, National Institute of Allergy and Infectious Diseases, National Institutes of Health, Bethesda, MD USA; Department of Transfusion Medicine, National Institutes of Health Clinical Center, Bethesda, MD USA; Sanaria Inc., Rockville, MD USA

## Abstract

**Background:**

In this phase 1 clinical trial, healthy adult, malaria-naïve subjects were immunized with radiation-attenuated *Plasmodium falciparum* sporozoites (PfRAS) by mosquito bite and then underwent controlled human malaria infection (CHMI). The PfRAS model for immunization against malaria had previously induced >90 % sterile protection against homologous CHMI. This study was to further explore the safety, tolerability and protective efficacy of the PfRAS model and to provide biological specimens to characterize protective immune responses and identify protective antigens in support of malaria vaccine development.

**Methods:**

Fifty-seven subjects were screened, 41 enrolled and 30 received at least one immunization. The true-immunized subjects received PfRAS via mosquito bite and the mock-immunized subjects received mosquito bites from irradiated uninfected mosquitoes. Sera and peripheral blood mononuclear cells (PBMCs) were collected before and after PfRAS immunizations.

**Results:**

Immunization with PfRAS was generally safe and well tolerated, and repeated immunization via mosquito bite did not appear to increase the risk or severity of AEs. Local adverse events (AEs) of true-immunized and mock-immunized groups consisted of erythaema, papules, swelling, and induration and were consistent with reactions from mosquito bites seen in nature. Two subjects, one true- and one mock-immunized, developed large local reactions that completely resolved, were likely a result of mosquito salivary antigens, and were withdrawn from further participation as a safety precaution. Systemic AEs were generally rare and mild, consisting of headache, myalgia, nausea, and low-grade fevers. Two true-immunized subjects experienced fever, malaise, myalgia, nausea, and rigours approximately 16 h after immunization. These symptoms likely resulted from pre-formed antibodies interacting with mosquito salivary antigens. Ten subjects immunized with PfRAS underwent CHMI and five subjects (50 %) were sterilely protected and there was a significant delay to parasitaemia in the other five subjects. All ten subjects developed humoral immune responses to whole sporozoites and to the circumsporozoite protein prior to CHMI, although the differences between protected and non-protected subjects were not statistically significant for this small sample size.

**Conclusions:**

The protective efficacy of this clinical trial (50 %) was notably less than previously reported (>90 %). This may be related to differences in host genetics or the inherent variability in mosquito biting behavior and numbers of sporozoites injected. Differences in trial procedures, such as the use of leukapheresis prior to CHMI and of a longer interval between the final immunization and CHMI in these subjects compared to earlier trials, may also have reduced protective efficacy. This trial has been retrospectively registered at ISRCTN ID 17372582, May 31, 2016.

**Electronic supplementary material:**

The online version of this article (doi:10.1186/s12936-016-1435-y) contains supplementary material, which is available to authorized users.

## Background

Despite significant reductions in the prevalence of malaria during the last 15 years [1], emerging drug and insecticide resistance and the significant ongoing burden of morbidity and mortality emphasize the need for an effective malaria vaccine. Such a vaccine is possible, as radiation-attenuated sporozoites (RAS) administered intravenously (IV) to mice [2] or by mosquito bite [3] to mice and non-human primates [4] induce almost complete sterile protection. During the 1970s, 1980s and early 1990s a series of human studies using *Plasmodium falciparum* RAS (PfRAS) delivered by bite of irradiated mosquitoes similarly induced nearly 100 % sterile protection as long as sufficient numbers of immunizing bites were administered [5–9]; since parasitaemia was completely prevented in these volunteers, all clinical manifestations of malaria were avoided. Beginning in 1989, additional human subjects were immunized with PfRAS and the immunological outcomes were extensively published [10–14]. Ten out of ten subjects (100 %) given greater than 1000 bites were fully protected against controlled human malaria infection (CHMI) conducted less than 10 weeks after immunization (one undergoing CHMI at 10 weeks was not protected), six of six (100 %) were protected on repeat CHMI within 10 weeks of primary CHMI, and five of six (83 %) were protected on repeat CHMI within 23–42 weeks of primary CHMI, indicating that protection was durable for at least 10 months [15]. These studies also showed that protection extended to heterologous strain parasites (parasites genetically and antigenically different from the immunizing strain), as several subjects immunized with an African malaria strain (NF54) were protected against a parasite cloned from a Brazilian isolate (7G8) [15].

Although these studies provided proof of concept that sporozoites could induce high-level immunity, as a vaccine for human use, PfRAS immunization was deemed impractical for many decades due to the complexity of administering a vaccine via mosquito bite, the requirement for a secure insectary and a laboratory for maintaining *P. falciparum* in culture, and the perceived need for five or more immunization sessions to achieve a sufficient number of bites. Recently, it has been demonstrated that the Sanaria PfSPZ vaccine, composed of aseptic, purified, cryopreserved, PfRAS is safe, well-tolerated, easily administered by syringe using a variety of routes, and can induce 100 % protective efficacy against CHMI when administered intravenously [16, 17]. PfRAS immunization by mosquito bites or by syringe therefore serves as a model for high-grade, cross-strain protective immunity in animals and humans, creating a strong rationale to develop a sub-unit vaccine approach that might provide equivalent protection, if the protective immune mechanisms and targeted antigens could be identified.

The sterile immunity induced by RAS appears to be mediated primarily by CD8+ and CD4+ T cell-dependent mechanisms targeting antigens expressed by sporozoites and liver-stage parasites [11, 13, 14, 18]. Responses to a liver-stage antigen were also identified in one study [14]. In rhesus monkeys, and in most murine studies, CD8+ cells were required for protection [19–22]. Murine studies suggest that inflammatory cytokines such as interferon-gamma (IFN-γ) induce the nitric oxide pathway in hepatocytes to kill liver-stage parasites [23], or that the infected hepatocytes are destroyed by direct cytotoxic activity [24]. Sporozoite-neutralizing antibodies likely contribute significantly to protection [10, 25], particularly when CHMI is conducted soon after immunization.

Antibodies also appear to contribute to protection. Studies in mice and humans show that immunization with RAS induces sporozoite-neutralizing antibodies [10, 16] that recognize the circumsporozoite protein (CSP), an abundant protein forming the surface coat of the sporozoite [4]. This finding led to the cloning of *P. falciparum* CSP [26, 27] and the formulation of several CSP-based sub-unit vaccines designed to induce protective antibodies [28, 29]. Although efficacy was low, subsequent development of CSP using a particle-based approach has led to the currently most advanced malaria sub-unit vaccine, RTS,S/AS01, that elicits 30 % protection in young children [30] primarily mediated by anti-CSP antibodies and CD4+ T cells [31, 32]. When tested in the field, RTS,S does not induce sterile protection, but rather reduces the frequency of clinical malaria. The lack of CD8+ T cell responses may be one reason. In addition to protein-based sub-unit vaccines, gene-based vaccines have shown promise, particularly in heterologous prime-boost regimens [33, 34], although efficacy is still well below that of RAS. The partial efficacy of these first generation sub-unit vaccines suggests that a better understanding of RAS-induced protective mechanisms may provide a rationale to develop alternative or improved sub-unit strategies using newly discovered antigens or more potently inducing cell-mediated immunity.

To address this objective, a new human trial using *P. falciparum* (strain NF54) RAS was conducted from 1999 to 2002 with the primary goal to obtain samples for investigation of protective immune mechanisms and antigen discovery. Fifteen healthy adult subjects were immunized five to six times by mosquito bite at intervals of 5–7 weeks (with the exact timing based on the availability of sufficient numbers of PfRAS-infected mosquitoes) to achieve a total of at least 1000 bites of irradiated, infected *Anopheles stephensi* mosquitoes, and seven received a similar number of non-infectious bites (mock-immunized controls) to control for the effects of mosquito salivary antigens injected during the mosquito bites. Ten of the true-immunized subjects and five non-immunized infectivity controls underwent CHMI with homologous strain (NF54) infectious sporozoites administered by five mosquito bites, to assess protection and to investigate possible correlation between the CHMI outcome and immune responses. Sera, plasma and peripheral blood mononuclear cells (PBMC), using leukapheresis to obtain large numbers of lymphocytes, were collected from all subjects. These samples have already been used to investigate the antigenic targets induced by PfRAS [35–40], underscoring the usefulness of these studies for vaccine development. Here, the safety, tolerability, protection, and humoral response data collected during this clinical trial are reported. The trial increases from 14 to 24 the total number of subjects receiving more than 1000 infectious bites and undergoing CHMI within the published literature.

## Methods

### Objectives

The objectives of this study were to determine whether a minimum of 1000 bites of irradiated *P. falciparum*-infected mosquitoes was safe and well tolerated and would elicit protection in up to 100 % immunized subjects as previously proposed [15], and to provide immune samples to investigate correlates of protection, including both the immunological responses and the targeted *P. falciparum* antigens. Research subjects were enrolled into three groups: (1) ‘true-immunized’ subjects who were immunized by the bites of *An. stephensi* mosquitoes infected with irradiated sporozoites, followed by CHMI; (2) ‘mock-immunized’ subjects who were immunized by the bites of uninfected mosquitoes, but not exposed to CHMI; and, (3) infectivity controls who were not immunized but were exposed to CHMI at the same time as the true-immunized subjects in order to prove the viability of the CHMI.

### Ethics

The study was conducted at the Naval Medical Research Center (NMRC) Clinical Trials Center between 1999 and 2002. At the time of these studies, the Food and Drug Administration (FDA) did not require the administration of infectious mosquito bites to be conducted under an Investigational New Drug (IND) allowance. This trial was retrospectively registered at ISRCTN ID 17372582. The study protocol was reviewed and approved by the NMRC Institutional Review Board in compliance with all federal regulations governing the protection of human subjects. Walter Reed Army Institute of Research (WRAIR) holds a Federal-wide Assurance from the Office of Human Research Protections (OHRP) under the Department of Health and Human Services as does NMRC. NMRC also holds a Department of Defense/Department of the Navy Federal-wide Assurance for human subject protections. All key personnel were certified as having completed mandatory human research ethics education curricula and training under the direction of the WRAIR Institutional Research Board or the NMRC Office of Research Administration (ORA) and Human Subjects Protections Program (HSPP). All potential study subjects provided written, informed consent before screening and enrolment and had to pass an assessment of understanding.

### Study population

Healthy, malaria-naïve, non-pregnant adults between the ages of 18 and 50 were included in this study. Malaria-naïve status was confirmed by travel history, medical history and *P. falciparum* CSP ELISA screening.

### True and mock immunization procedures

The infected mosquito batches used for true-immunization were infected 14–21 days prior to human biting with the chloroquine-sensitive NF54 strain of *P. falciparum* by membrane feeding on in vitro blood cultures at the Biological Research Institute, Rockville, MD, USA. Monitoring for salivary gland infections was conducted by hand dissection of a representative sample from the batch, grading infection rates as gland scores: 1–10 sporozoites = gland score 1; 11–100 sporozoites = gland score 2; 101–1000 sporozoites = gland score 3; and >1000 = gland score 4. A gland score of two or higher was used as the cut-off to count a mosquito as ‘infected’, although those with ten or fewer sporozoites on dissection could still be infectious and inject sporozoites during feeding. The morning of an immunization procedure, mosquito batches with 70 % or more of mosquitoes showing gland score 2 or higher were transported to NMRC/WRAIR, Silver Spring, MD, USA and subjected to 15,000 cGy using a Model 109-68 Cobalt^60^ irradiator.

Both true- and mock-immunizations were conducted in the secure WRAIR/NMRC insectary by placing two cylindrical cardboard containers with mosquito netting at one end, each holding approximately 200 mosquitoes, in contact with the volar surface of one forearm for 5 min, followed 2 min later by a second 5-min feed with the same mosquitoes at the same sites. Consistent with previous experience, approximately 70 % of the 400 mosquitoes in the two containers (200 × 2) took a blood meal. After the two 5-min feeding sessions, a sample of the engorged mosquitoes was hand-dissected to calculate infectivity rates. The total number of engorged mosquitoes was multiplied by the per cent of mosquitoes with mean gland grade at least 2 to estimate the dose of infectious bites. The goal for the full immunization series was for the true-immunized group to receive a minimum of 1000 irradiated infectious mosquito bites before CHMI. In practice, this required five to six immunization sessions. Similarly, the goal for the mock-immunized group was to receive a minimum of 1000 non-infectious mosquito bites, with the number of bites from each immunization session calculated in this latter case as the number of engorged mosquitoes. Mosquitoes used for mock immunization were raised, handled and irradiated in the same fashion as those for true immunization except they were not fed on *P. falciparum* blood cultures. Both true- and mock-immunized subjects were observed on site for at least 30 min after each immunization.

### Controlled human malaria infection (CHMI)

Five non-irradiated mosquitoes, infected with the same NF54 strain of *P. falciparum* used for immunization were allowed to feed once for 5 min on the subjects. All fed mosquitoes were dissected to determine the infectivity rate. Replacement mosquitoes for those of the initial five not feeding or feeding but found on dissection to have gland grades of 1 or less (ten sporozoites or fewer) were then allowed to feed and this process was repeated until five infectious bites had been achieved. Beginning 7 days after CHMI, subjects were assembled each night in a regional hotel for clinical monitoring by study staff. Each morning, thick blood smears were made for microscopic examination, and sufficient passes over the slide were made using the high-power objective, such that approximately 40 µL of blood were examined. The presence of two parasites was required for a positive diagnosis, leading to immediate anti-malarial treatment with chloroquine phosphate. The treatment regimen was directly observed and included 1000 mg chloroquine phosphate salt (600 mg chloroquine phosphate base) immediately, 500 mg salt (300 mg base) at 6 h and again at 24 and 48 h. Subjects who were positive were monitored daily by symptom checks and blood smears until three consecutive negative smears were documented and subjects remaining negative were similarly monitored until day 21 post CHMI, then approximately every other day until day 28. Those remaining negative on day 28 were considered fully protected.

### Adverse events (AEs)

Subjects were examined by physical examination and verbal questioning for local adverse events at 24, 48 and 72 h and at 1 and 2 weeks after each immunization. Although specific systemic symptoms were not actively solicited, subjects were asked in open-ended fashion to describe any systemic symptoms to the evaluating clinician, and these were recorded.

### AE grading

Local AEs were subjectively graded as follows:

*Mild*: Minimally apparent symptoms noticed by the study subject (pain, tenderness, pruritus) or signs noticed by the examiner (erythaema, induration, swelling, lymphadenitis) but not requiring treatment.

*Moderate*: Symptoms or signs quite evident to the study subject (pain, tenderness, pruritus) or the examiner (erythaema, induration, swelling, lymphadenitis), potentially interfering with the activities of daily living (ADLs); treatment offered (i.e., study subject provided with topical corticosteroid cream to apply as needed).

*Severe*: Clinically significant findings interfering with daily activities; study subject requested or examiner recommended immediate local and/or systemic treatment with topical corticosteroids and/or oral antihistamines/corticosteroids/non-steroidal anti-inflammatory drugs.

Systemic AEs were subjectively graded as follows:

*Mild*: No treatment required; ADLs not compromised (subject able to work, or attend school).

*Moderate*: Outpatient treatment required, ADLs only minimally compromised (subject able to work, or attend school). *Severe*: Outpatient treatment required, ADLs compromised (subject not able to work or attend school). *Serious:* AEs resulting in death; AEs that were life-threatening, meaning that failure to intervene could result in hospitalization or death (example: bronchospasm requiring parenteral medication in the emergency room, or grand mal seizure evaluated in the emergency room but not resulting in hospitalization); AEs leading to or prolonging inpatient hospitalization; AEs resulting in persistent or significant disability or incapacity, including addiction; congenital anomaly or birth defect in an infant conceived by a subject.

### Laboratory tests

Screening clinical laboratory tests were initially collected to determine enrolment eligibility. These tests included a complete blood count (CBC) and screens for hepatitis B virus, hepatitis C virus and human immunodeficiency virus. Once a study subject was enrolled but prior to immunization, additional sampling was performed by withdrawal of whole blood and by leukapheresis, to provide pre-immunization serum, PBMCs and plasma for banking. Additional blood collections were performed at various time points throughout the trial for banking serum, PBMCs and plasma. Leukapheresis was repeated halfway through the immunization series in some subjects and after the final immunization/prior to CHMI in all subjects that underwent CHMI. There was no systematic collection of safety laboratory data beyond screening for enrolment and a CBC prior to each leukapheresis. Additional samples for safety laboratory tests were collected only in study subjects as clinically indicated.

### Immunofluorescence antibody assay (IFA) using sporozoites

Serum antibody levels were assessed by IFA against air-dried *P. falciparum* 3D7 strain sporozoites; 3D7 is a clone of NF54 obtained by limiting dilution [41]. To prepare the IFA slides, infected mosquitoes were suspended in 3 % bovine serum albumin (BSA) at a concentration of 10^6^ sporozoites per mL. An aliquot of 10 µL containing 10^4^ sporozoites was delivered into each well of the antigen slide. The antigen slides were allowed to air dry at room temperature and were kept at −70 °C until used. 20 µL of a twofold serial dilution of test or control serum in PBS containing 2 % BSA was added to each well of the antigen slides. The slides were incubated for 1 h at 37 °C, washed three times in PBS, 5 min each wash. Each well was incubated for 30 min at 37 °C with 20 µL of a 1:50 dilution of FITC-labelled goat anti-human IgG (H^+^L) (Kirkegaard and Perry). The slides were washed again, mounted in a Vectashield mounting medium (Vector Laboratories, Inc.) and examined under an Olympus UV microscope and end-point titers were determined as the last dilution above the background that fluorescent parasites were observed.

### Enzyme-linked immunosorbent assay (ELISA)

The *P. falciparum* recombinant proteins used in the ELISA assays, CSP, SSP2/TRAP, EXP1, and LSA1 have been previously described [42–44]. Stock solutions of *P. falciparum* recombinant proteins were diluted in phosphate buffered saline, pH 7.2, to the optimal concentration of each (0.5 µg/mL for CSP, 1.0 µg/mL for SSP2/TRAP, 2.0 µg/mL for EXP1, 4.0 µg/mL for LSA1) as previously described [45, 46]. The ELISA titre was defined as the calculated serum dilution yielding an optical density of 0.5 in the assay. Samples were considered positive if the titre of the sample post-immunization was greater than the titre plus two standard deviations of the sample pre-immunization and greater than twofold higher than the corresponding pre-immunization sample.

### Sample size and statistical assessment

The primary objective of the study was to collect PBMCs, sera and plasma before and after PfRAS immunization, and then to characterize protective immune responses and identify protective antigens for malaria vaccine development by comparing protected and non-protected research subjects. The number of study subjects was constrained by the capacity to generate infected mosquitoes.

The log rank test was used to compare time to parasitaemia between infectivity control and non-protected immunized subjects. The Mann–Whitney U test was used to compare the interval between protected and unprotected subjects and the interval between leukapheresis and CHMI for protected and non-protected subjects. The repeated measure analysis of variance was used to compare the means of the IFA titres between protected and non-protected subjects. The IFA titres were log10 transformed prior to the analysis. The repeated measure analysis of variance was also used to compare the means of the ELISA titres between protected and non-protected subjects. Statistical significance was defined as a two-tailed P ≤ 0.05.

## Results

### Study flow

**Fig. 1:**
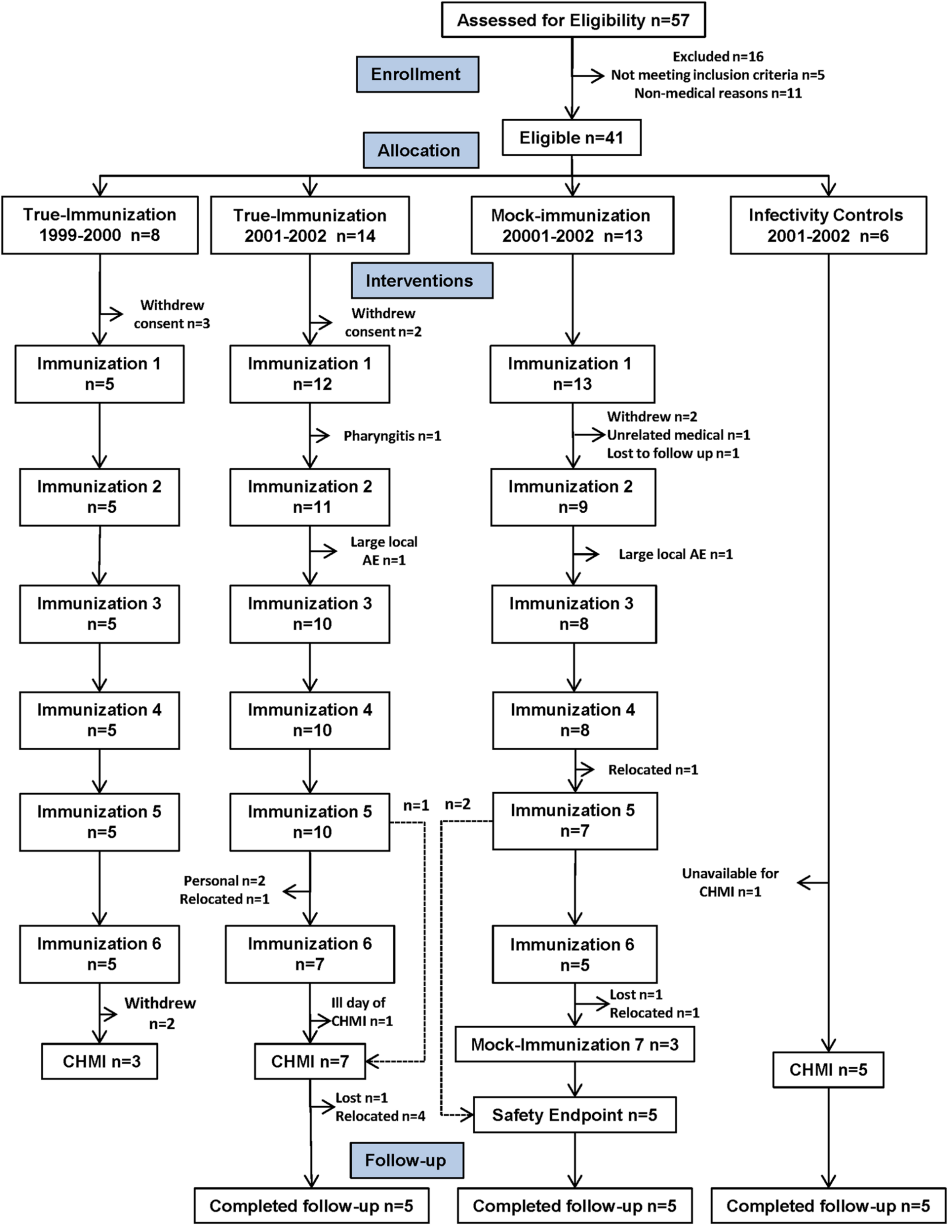
Flow diagram of immunized and control subjects. 41 subjects met all eligibility criteria and were assigned to the true-immunization group (n = 22), mock-immunization group (n = 13) and infectivity controls (n = 5). True-immunized group contained subjects enrolled in 1999–2000 and 2001–2002

The participant flow is shown in Fig. 1. Recruitment took place at the NMRC Clinical Trials Center between September 1999 and August 2002. A total of 57 subjects were assessed for eligibility and 16 were excluded. The remaining 41 subjects, who met all screening criteria, were enrolled and assigned to the true-immunized (22 subjects), mock-immunized (13 subjects) and infectivity control (six subjects) groups. Thirty of these 41 enrolled subjects initiated the immunization regimens (17 true-immunized, 13 mock-immunized), and three true-immunized underwent CHMI in 1999–2000 and seven true-immunized underwent CHMI in 2001–2002. Demographics of the study subjects are shown in Table 1. Immunization was performed when PfRAS were available resulting in varying immunization schedules for the subjects, and the CHMI was performed in two groups as described below.

**Table 1:** Study subjects demographics

	True-immunized		Mock-immunized	Infectivity controls
	1999–2000	2001–2002		
Gender				
Male	5	12	11	4
Female	0	0	2	1
Total	5	12	13	5
Age (mean + SD)	42 ± 10	34 ± 8	34 ± 7	36 ± 8
Race/ethnicity				
African–American	0	3	1	2
Caucasian	5	8	7	2
Hispanic	0	0	0	0
Not known	0	1	5	1
Total: true-immunized		17		
Total: mock-immunized			13	
Total: infectivity controls				5

Seventeen subjects were enrolled into the true-immunized group that received bites of PfRAS mosquitoes; five subjects were enrolled in 1999–2000 and 12 subjects were enrolled in 2001–2002. Thirteen subjects were enrolled into the mock-immunized group that received bites of uninfected mosquitoes, and six subjects enrolled as infectivity controls of whom five received CHMI

### Immunization procedures

*True-immunized group*: Five subjects enrolled in 1999–2000 completed six immunizations and three subjects received CHMI in the first cohort in 2000. Ten of 14 subjects enrolled in 2001–2002 completed five immunizations, and seven of these ten subjects completed six immunizations; six subjects who completed six immunizations and one subject who completed five immunizations (total seven subjects) received CHMI in the second cohort in 2002 (Fig. 1). Three of the ten subjects who received five immunizations withdrew after the fifth immunization (one for personal reasons; one because of a family emergency, and one due to relocation outside the geographic area). One of the seven subjects who completed six immunizations did not receive CHMI because of an unrelated illness on the day of CHMI. In addition, one subject was withdrawn after the first immunization due to illness, and one subject was withdrawn after the second immunization (see “AEs” section).

**Fig. 2:**
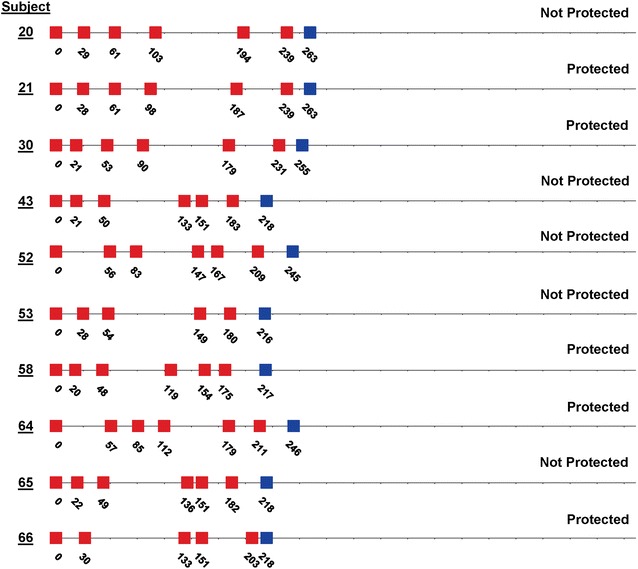
Immunization regimes for the ten subjects who underwent CHMI. *Red boxes* indicate PfRAS immunization, *blue boxes* represent CHMI and the numbers indicate the study day for that subject (day 0 was the day of the first immunization). Subjects 20, 21 and 30 were in the first cohort for CHMI, and the rest were in the second cohort for CHMI

The immunization regimens for the ten subjects who underwent CHMI are shown in Fig. 2, and summarized in Table 2. Although the numbers of subjects in these two groups were too small to reliably distinguish statistically, the ranges of numbers of bites, numbers of immunizations, intervals between immunization, intervals before CHMI, and salivary sporozoite gland score appeared similar, suggesting that these parameters were highly similar between the two groups. When combined, the median number of infectious bites per immunization session was 214 (range 175–260), the median total number of infectious bites was 1247 (range 1005–1561), and the median interval between immunizations was 43 days (range 35–48 days).

**Table 2:** Summary of immunizations in this study subjects who were true-immunized in 1999–2000) and 2000–2002

	Group	
	1999–2000	2000–2001
Number of subjects	3	7

	Median	Range	Median	Range
Infected bites in the first immunization	148	138–334	227	178–252
Total infected bites	1408	1322–1561	1161	1005–1561
Median infected bites per immunization	235	220–260	212	175–260
Number of immunizations	6	6–6	6	5–6
Duration of immunizations (days)	239	231–239	183	175–211
Median immunization interval (days)	37	21–91	29.5	15–100
Interval before CHMI (days)	24	24–24	35.5	15–42
Median gland grade score^a^ during immunization	3.8	3.8–3.9	3.4	3.2–3.6
Median gland grade score during CHMI	2.4	2.4–2.8	2.8	2.4–3.2

The median and range of the immunizations are shown of all subjects in the two groups (1999–2000 and 2000–2001) who underwent CHMI. Sample sizes were too small to reliably do a statistical comparison

^a^ *Gland score* as defined in “Methods” section, salivary infection rates were defined as gland scores: 1–10 sporozoites = gland score 1; 11–100 sporozoites = gland score 2; 101–1000 sporozoites = gland score 3; and >1000 = gland score 4

*Mock-immunized group*: In parallel to these true immunizations, seven subjects received at least five mock immunizations over 175–239 days (Fig. 1) receiving a total of 1210–1890 (median 1636) irradiated non-infectious mosquito bites. None of these subjects underwent CHMI.

*Additional true-immunized subjects*: Four of the ten subjects who underwent CHMI, including three from the 1999–2000 group and one from the 2001–2002 group, and one subject enrolled in 1999–2000 who was immunized but did not undergo CHMI, received further RAS immunizations (see Additional file 1). One of the four subjects immunized and undergoing CHMI with the 1999–2000 cohort, subject number 20, was not protected, was immunized with six additional immunizations, and joined the second cohort CHMI, and was protected; he received one further immunization following the second CHMI, for a total of 13 immunizations (see Additional file 1). The several immunizations described here that were not followed by CHMI contributed to the safety data presented below.

### Safety and tolerability

#### Local adverse events (AEs)

**Fig. 3:**
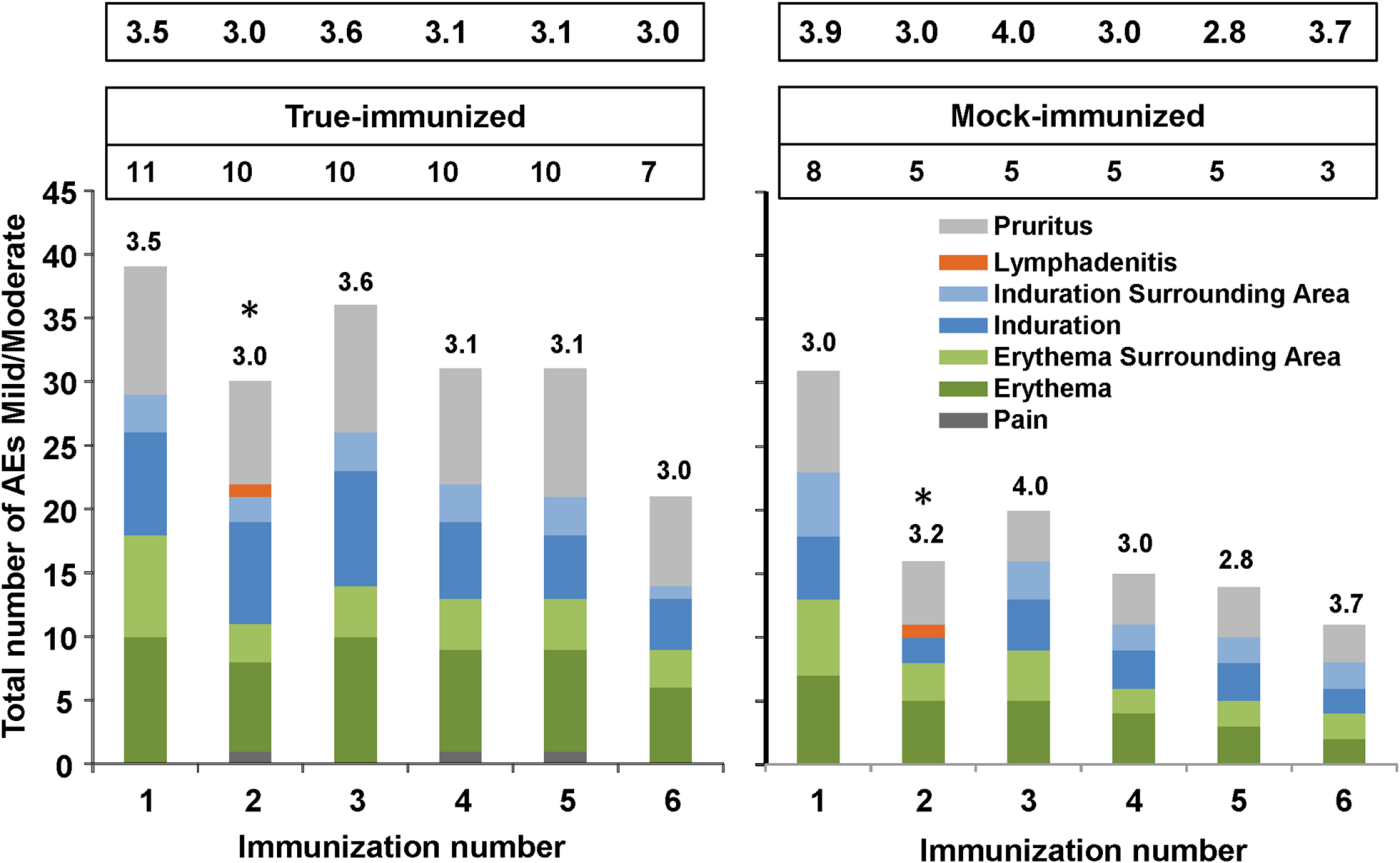
Frequency of true- and mock-immunized subjects experiencing local adverse events after each immunization 1–6. Number of local AEs (mild/moderate) experienced by each subject after true- and mock-immunizations 1–6. The numbers at the *top* of the* graphs* represent the number of individuals immunized at each immunization. The numbers at the * top* of each* column* represent the number of AEs/subject. The number of AEs per subject per immunization for each group were very similar in the true-immunized and mock-immunized subjects. All AEs were mild or moderate in severity except for one severe AE (swelling) in one true- and one-mock immunized subject at immunization 2 denoted by *asterisks*

During the interval between study completion and publication, these records were securely maintained in a storage facility that flooded; this incident resulted in the loss of several original records. Original subject records, source documents and case report forms (CRFs) were available for 19 of 30 immunized (11 true- and eight mock-immunized) subjects that received immunization 1, for 15 subjects for immunizations 2–5 (ten true- and five mock-immunized), and ten subjects who received immunization 6 (seven true- and three mock-immunized subjects). The frequency of AEs during immunizations 1–6 is shown in Fig. 3. The remaining 11 subjects with missing source data comprised seven subjects who received at least one true-immunization and four who received at least one mock-immunization. However, AE summary data from these 11 subjects were available in reports submitted to the IRB during the conduct of the trial. Comparing these summary reports on 30 subjects to the available original source documentation on 19 subjects indicated that no severe or serious AEs were omitted from the missing data set. While mild and moderate AEs could not be enumerated individually for the 11 subjects with missing charts, the findings of mild and moderate AEs on the 19 subjects with source documentation were representative of the total mild and moderate AEs as presented in the summary reports.

All immunization safety data from the available source documents of 19 subjects were analysed and the mild to moderate AEs, after each immunization, are shown in Fig. 3. In general, most AEs occurred within the first 48 h and resolved in seven to 14 days. Subjects noted mild discomfort during mosquito feeding and experienced mild to moderate local reactogenicity afterwards. Immediately after each 10-min biting session, the skin exhibited mild to moderate erythaema restricted to the circular area in contact with the cylindrical mosquito container, with or without associated mild to moderate induration and swelling. In many subjects, small erythaematous papules demarcated the precise sites of mosquito probing; these were also confined to the region where the mosquito container was applied. In some individuals, the papules coalesced into an elevated, confluent, erythaematous plaque. These plaques as well as other signs and symptoms typically resolved within 24–72 h. Pain and tenderness represented only a small proportion of the reported AEs while pruritus and erythaema were more common. Because subjects received varying numbers of immunizations in sessions 1–6, the numbers of AEs/subject are shown, and were similar after each immunization (true-immunized geometric mean 3.2 AEs per subject per immunization, range 3.0 to 3.6; mock-immunized geometric mean 3.4 AEs per subject per immunization, range 2.8–4.0).

In two subjects, local reactions extended significantly beyond the area of skin to which the container of feeding mosquitoes had been applied, and were graded as severe. These occurred following the second immunization in two subjects, one true-immunized (subject #29) and one mock-immunized (subject #35), who developed “large local reactions” consisting of swelling that extended from the wrist to the elbow (see asterisk in Fig. 3). Interestingly, the mock-immunized subject had shown erythaematous lymphangitic streaks on the volar aspect of the arm from the bite site to the axillae after the first immunization. The clinical investigators obtained a consultation from a clinical immunologist and allergist who advised that the reactions were likely due to IgE-mediated histamine responses following the introduction of mosquito salivary antigens. In both cases, the large local reactions resolved uneventfully over 1or 2 days without sequelae. Both subjects were withdrawn from further participation and were not re-immunized. One was available for follow-up and reports that during the subsequent 10 years, mosquito bites often result in an immediate wheal and flare reaction (1 cm in diameter) with itching and irritation, resolving over 10 min, but no recurrence of the large local reaction.

#### Systemic adverse events

**Fig. 4:**
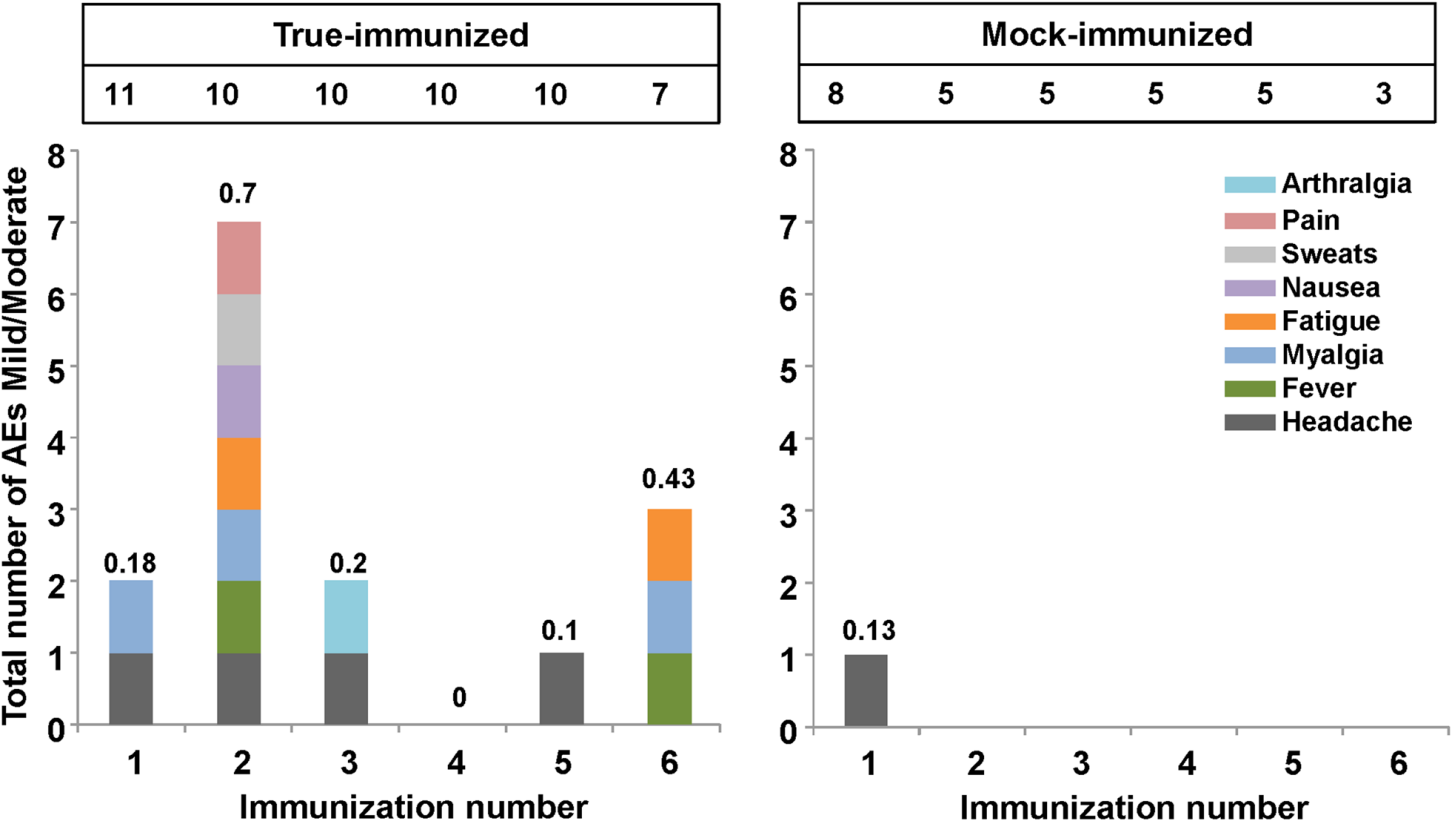
Frequency of true- and mock-immunized subjects experiencing systemic adverse events after each immunization 1–6. Number of local AEs (mild/moderate) experienced by each subject after true- and mock-immunizations 1–6. The numbers at the *top* of the* graphs* represent the number of individuals immunized at each immunization. The numbers at the *top* of each* column* represent the number of AEs/subject. The timing of each immunization is shown in Fig. 2

Systemic AE data were likewise available from the source documents of the same 19 immunized (11 true- and eight mock-immunized) study subjects for which local AEs are reported, and are provided in Fig. 4. In general, most systemic AEs occurred within the first 48 h and resolved within 7 days. Among true-immunized subjects, systemic AEs were generally characterized as mild and consisted of headache, myalgia, nausea, and low-grade fever. One subject reported a cough of moderate intensity following immunization, deemed unlikely related to immunization. In contrast, systemic AEs were absent after mock-immunization, except one mild AE (headache) in one subject after the first mock-immunization (Fig. 4). This difference suggests that *P. falciparum* sporozoites may elicit systemic AEs, although of lower frequency than local AEs.

Two true-immunized study subjects, number 20 and number 66, experienced unexpected systemic reactions that were unique to these subjects and are not therefore included among the AEs in Fig. 4. These individuals reported a range of symptoms ranging from mild to severe that, due to timing and similarity, appeared likely related to immunization. These systemic AEs are reported in Additional file 2.

#### Clinical laboratory adverse events

Clinical laboratory tests were performed on all subjects at screening, but thereafter only on a case-by-case basis in accordance with the clinical judgment of an investigator evaluating a complaint by a study subject. Twenty-two tests were conducted on 19 subjects at various time-points during the immunization phase of the trial (prior to CHMI). Due to the non-systematic collection of clinical laboratory samples, it is uncertain when most laboratory AEs occurred; however, the laboratory AEs resolved prior to receiving additional immunizations. For one subject, one post-immunization aspartate aminotransferase (AST) and alanine aminotransferase ALT) were slightly elevated, 67 (normal 17–49) and 62 (7–59), respectively; several CBC indices were abnormal in 11 subjects with the most out-of-range values a haemoglobin (HGB) of 12.5 (normal 14–18), haematocrit test (HCT) of 36.6 (normal 42–52), white blood cell (WBC) of 3.7 (normal 4.0–11.0), and red blood cell (RBC) of 4.04 (normal 4.7–6.1)]. These were all grade 1 excursions in severity based on the FDA’s toxicity grading scale for healthy adult and adolescent subjects enrolled in preventive vaccine clinical trials [47].

### Protective efficacy and time to parasitaemia

**Fig. 5:**
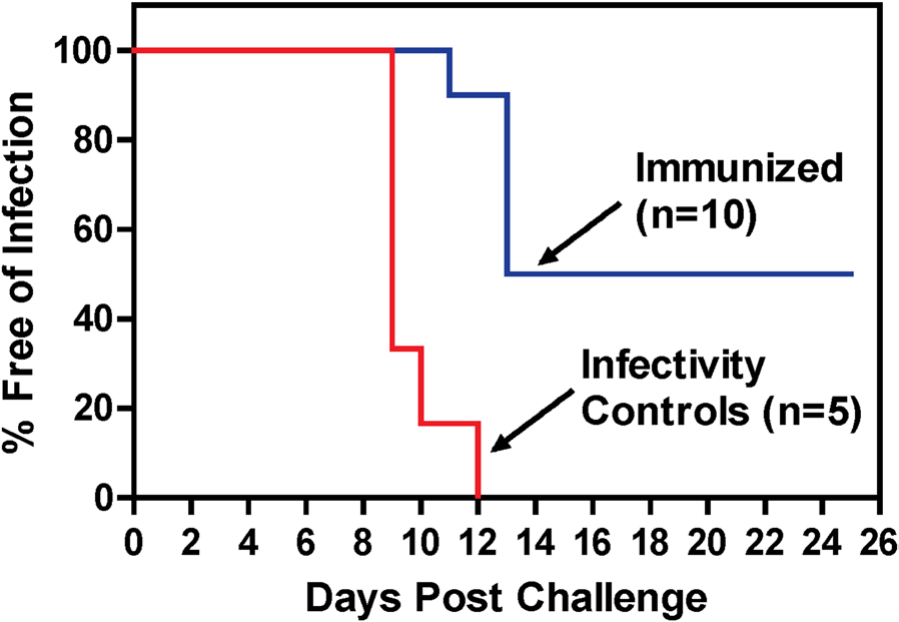
Development of parasitaemia in the immunized and infectivity control subjects. Parasitaemia-free survival curves (Kaplan–Meier) for immunized subjects and infectivity controls based on microscopic examination of peripheral blood smears. P < 0.0001 (Log rank test)

The three subjects enrolled in 1999–2000 received CHMI as one group, and seven subjects enrolled later received CHMI as the second group. Because the immunization schedule for many subjects was individualized (Fig. 2), the timing of CHMI varied from 15 to 42 days after the last immunization (Fig. 2). Five of ten subjects were sterilely protected after CHMI (2/3 in the first CHMI, 3/7 in the second CHMI). The time to parasitaemia was delayed in the five unprotected subjects compared to infectivity controls (13 vs 10 days, P = 0.03) (Fig. 5). Overall, the time to parasitaemia observed in the Kaplan–Meier plot in the true-immunized and infectivity controls was statistically significantly different (P < 0.0001, log rank test). The mock-immunized subjects did not undergo CHMI.

### Comparison of immunization procedures with prior studies: association between delayed CHMI and loss of protection

**Fig. 6:**
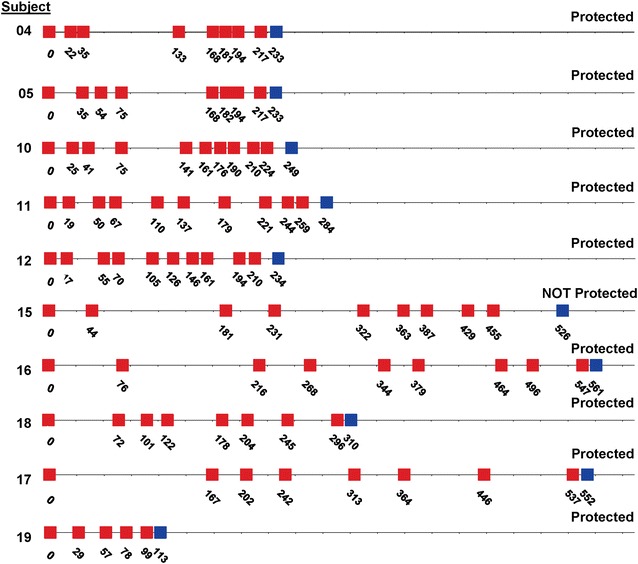
Immunization regimes for ten subjects who underwent CHMI in a prior study conducted 1989–1999. Ten subjects were immunized by bite of PfRAS during 1989–1999 [15] and received CHMI. The days of immunization as shown, beginning at day 0, and the numbers of infectious bites, are shown as *red boxes*. The day of CHMI is shown as *blue boxes*. Nine of 10 subjects were protected

The 50 % protection in this study is lower than that in previous trials [15]. The immunization procedures for ten subjects previously immunized in 1989–1999 at NMRC are shown in Fig. 6. Ten subjects received five to ten immunizations over 99–547 days, receiving a total of 1001–1163 (median 1070) PfRAS bites, and underwent CHMI 14–71 days later with five infectivity controls. Nine of these ten subjects were sterilely protected. The median number of infectious bites per immunization session was 125 (range 109–210) and the median of the estimated grades of salivary gland scores was 3.2 (range 3.0–3.7).

**Fig. 7:**
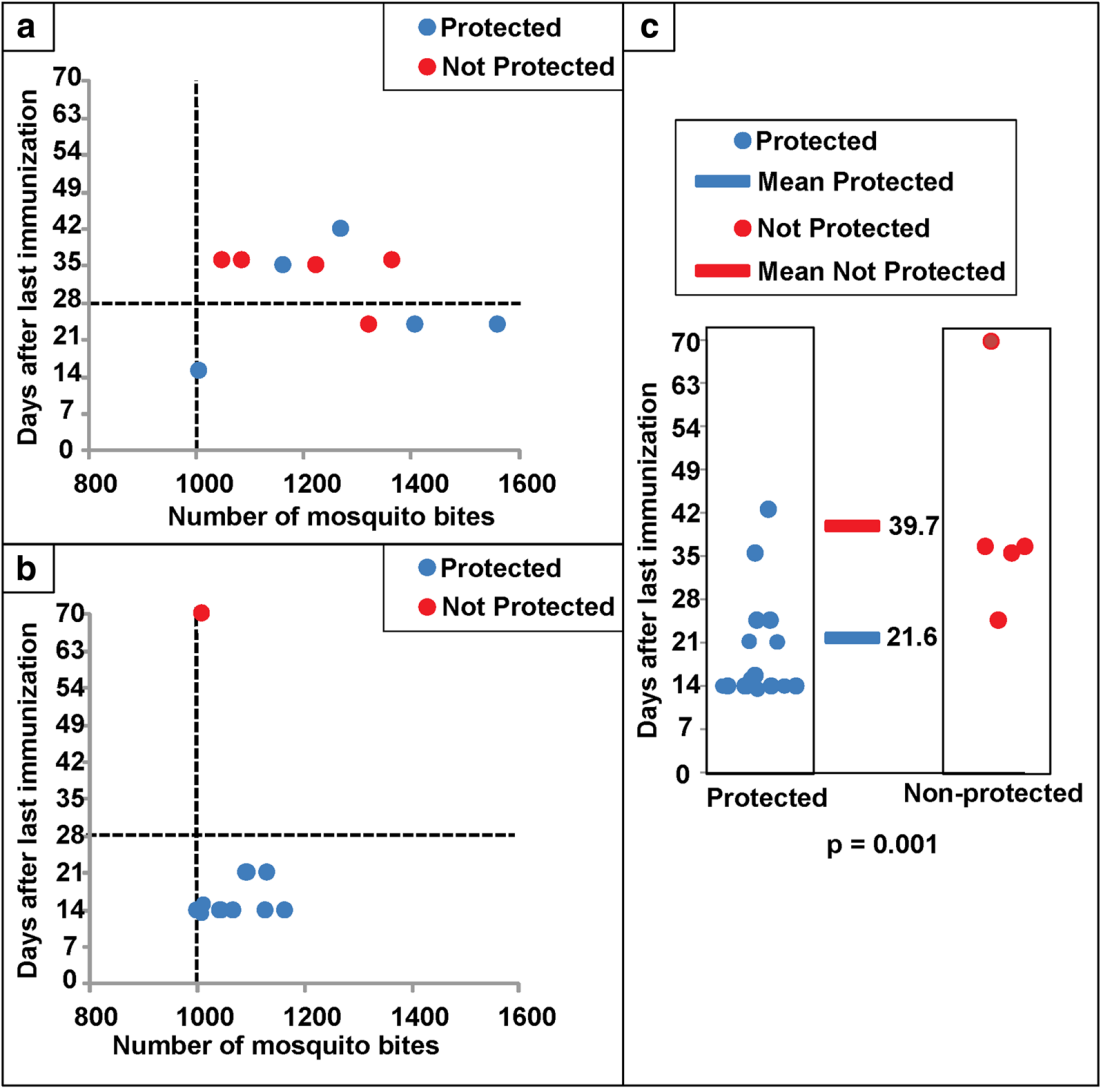
Comparison of the interval (in days) from last immunization to CHMI for individuals immunized in 1999–2002 (this study) and 1989–1999. The relationship between total number of mosquito bites and days after CHMI and protective efficacy is shown for the 1999–2002 study (**a**) and the 1989–1999 studies [15] (**b**). The threshold number that induced up to 93 % protection in previous studies [15] is indicated by a *dotted vertical line*. Protected subjects are shown as *blue dots*, and non-protected subjects are shown as *red dots*. For both studies, subjects were predominantly protected when CHMI was less than 28 days, and were predominantly not protected more than 28 days, after the last immunization, shown by the *horizontal dotted line*. However, these differences were not statistically significantly different in either study using the Mann–Whitney U test.** c** Protected and non-protected subjects in the 1999–2002 and 1989–1999 studies are combined, and grouped according to time of CHMI in days after the final immunization; the median time in days is shown for each group as colour-coded *horizontal lines*, and the difference was significant (P = 0.001) using Mann–Whitney U test

The immunization procedures for the current study (Table 2) are combined and compared to those for the 1989–99 study in Table 3. The 1989–99 volunteers received significantly (P = <0.05) fewer total number of infectious bites, fewer number of infectious bites in the first immunization, fewer bites per session, more immunization sessions, longer duration of immunization, shorter interval between last immunization and CHMI, decreased median gland score during immunization, and increased gland score during CHMI, but the median interval between immunizations was similar (Table 3). For both studies, subjects were predominantly protected when CHMI was less than 28 days, and were predominantly not protected more than 28 days, after the last immunization (Fig. 7). The times to CHMI between protected and non-protected volunteers were not statistically different in the current study. However, when the data from all 20 research subjects (current study and prior study) were combined, the median interval between last immunization and CHMI was significantly different between protected and unprotected subjects (20.0 vs 36.0 days; P = 0.01, Mann–Whitney U test; Fig. 7), suggesting that PfRAS induced protective immunity may have been short-lived.

**Table 3:** Summary of immunizations in this study (1999–2002) and the previous study (1989–1999)

A	Individual study data				P value
	1999–2002^a^		1989–1999		
	Median	Range	Median	Range	
Infected bites in the first immunization	224	138–334	148	130–210	0.0065
Total infected bites	1247	1005–1561	1092	1001–1163	0.0288
Median infected bites per immunization	214	175–260	125	109–210	0.0006
Number of immunizations	6	5–6	8.5	5–10	0.0014
Duration of immunizations (days)	206	175–239	241.5	99–547	0.0450
Median immunization interval (days)	43.5	35–48	31	23–77	0.2401
Median Interval before CHMI (days)	35	15–42	16	14–71	0.0434
Median gland grade score^a^ during immunization	3.5	3.2–3.9	3.2	3.0–3.7	0.0045
Median gland grade score during CHMI	2.7	2.4–3.2	3.2	2.8–3.4	0.0017

B	Combined data (1989–2002)				P value
	Protected		Non-protected		
	Median	Range	Median	Range	
Infected bites in the first immunization	159	130–334	148	133–250	0.4829
Total infected bites	1110	1001–1561	61170	1008–1366	0.7728
Median infected bites per immunization	138	109–260	125	112–228	0.2652
Number of immunizations	8	5–10	6	5–9	0.2293
Duration of immunizations (days)	221	99–547	196	180–455	0.3639
Median immunization interval (days)	38.5	23–77	43.5	36–57	0.3630
Median Interval before CHMI (days)	20	14–42	36	25–71	0.0088
Median gland grade score^b^ during immunization	3.3	3.0–3.9	3.5	3.0-3.8	0.2155
Median gland grade score during CHMI	3.1	2.4–3.4	2.7	2.4–3.2	0.0629

*Part A* summaries (median, range) of the immunizations in the 1999–2002 and 1989–1999 [15] studies were compared using the Mann–Whitney U test, where significance is P ≤ 0.05; these parameters were significantly different between each study except the median time between each immunization

*Part B* summaries of protected and non-protected subjects in each study were combined and compared using the Mann–Whitney U test, where significance is P ≤ 0.05; only the interval before CHMI (days between last immunization and CHMI) was significant

^a^Derived from combining the two groups shown in Table 2

^b^ *Gland score* as defined in “Methods” section, salivary infection rates were defined as gland scores: 1–10 sporozoites = gland score 1; 11–100 sporozoites = gland score 2; 101–1000 sporozoites = gland score 3; and >1000 = gland score 4

### Effect of leukapheresis and protection

Leukapheresis was performed in the 1999–2002 study but not the 1989–1999 study. Leukapheresis may temporarily remove a significant proportion of circulating PBMC [48] that might affect T cell-dependent protection. Liver-resident T cells are thought to mediate protection [49], and the effects of leukapheresis on this population are not known. However, there was no association between the timing of leukapheresis since the subjects with the longest interval were not protected, while those with the shortest interval were protected (P = 0.40, Mann–Whitney U test) (see Additional file 3).

### Repeat CHMI

Subject number 20, as previously discussed under AEs following immunization #13, was the only subject to undergo a repeat CHMI in this study (see Additional file 1). He received six true-immunizations for a total of 1322 infectious bites (estimated) as part of the first CHMI cohort. He underwent CHMI and was not protected. Subsequently, his enrolment continued and he received an additional six true-immunizations as part of the second CHMI cohort for a total of 12 immunizations. He underwent a secondary CHMI as part of Cohort 2 and was protected. He later received a 13th immunization that was associated with the systemic AE, which is also described in Additional file 2.

### Humoral responses

**Fig. 8:**
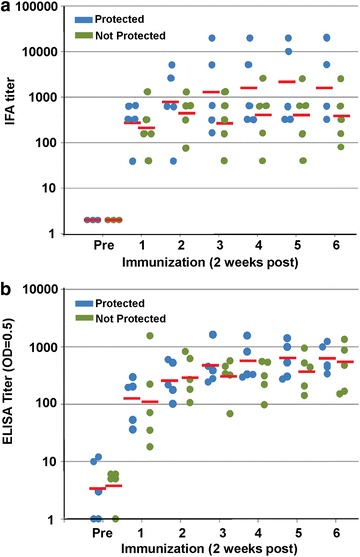
IFA and ELISA CSP responses. IFA (**a**) and ELISA (**b**) titres measured at pre-immunization (Pre) and 2 weeks after each immunization. *Blue circles* represent protected subjects, *green circles* represent non-protected subjects, and the *red line* represents the geometric mean of each group. There was no significant difference between protected and non-protected subjects

Peak sporozoite IFA and CSP ELISA titres generally occurred between the second and fourth immunizations (Fig. 8) but the difference in geometric mean titres between protected and non-protected subjects was not statistically significant. Low titres (<1:1000) of anti-TRAP antibodies were detected in 8/10 subjects but there were no detectable antibodies to LSA1, Exp1 or LSA3 in any subject.

## Discussion

Immunization with RAS by mosquito bite established the original gold standard for protection against falciparum malaria in the early 1970s, and earlier studies of PfRAS showed up to 93 % efficacy (13/14 subjects) [15]. These studies established that a malaria vaccine was feasible, and that sporozoite-specific antibodies and CD4+ and CD8+ T cells were induced by this form of immunization. Murine and simian studies established that CD8+ cells were required for protection [19–21]. PfRAS administered by mosquito bite is not being developed as a human vaccine, although considerable progress is being made using radiation-attenuated, aseptic, purified, cryopreserved sporozoites (PfSPZ Vaccine) administered by direct venous inoculation using a syringe [16, 17]. The study reported here was undertaken to further elucidate the mechanisms and antigenic targets of protective immunity induced by immunization with PfRAS by mosquito bite through the collection of sera and PBMCs from protected and non-protected subjects. The results of several studies using the sera and PBMCs from this clinical study have been published [35–37, 50, 51]. Here, the method of immunization, safety and tolerability, and antibody responses to selected *P. falciparum* antigens are reported.

In earlier PfRAS studies, including a few subjects that received *Plasmodium vivax* RAS, immunization was safe and well tolerated, highlighting as common events mild discomfort during mosquito feeding, erythaema, erythaematous papules, focal and sometimes more generalized local swelling, mild headaches and malaise that spontaneously resolved within 24 h [15]. In the current study, PfRAS immunization was also generally well tolerated, although two subjects experienced significant large local reactions of the forearm (swelling from elbow to wrist) and two other subjects experienced sudden onset systemic symptoms 16 h after immunization. Due to concern regarding the potential for systemic allergic reactions if immunizations were to continue, both cases of large local reaction led to exclusion from further participation. Other than the two large local reactions, local AEs were consistent with prior reports and with reactions to mosquito bites in nature: erythaema, papules, swelling, and induration. Large local reactions have only rarely been reported following mosquito bites in nature [52] and may relate to the very large antigenic load associated with hundreds of mosquito bites occurring simultaneously during immunization sessions. Because one of the two subjects experiencing a large local reaction to the mosquito bites was mock-immunized and that reaction was very similar to that of the true-immunized subject, it is probable that sporozoites themselves did not contribute to the large local reaction in the true-immunized subject.

Systemic AEs occurred at a lower rate than local AEs and were generally mild, consisting of headache, myalgia, nausea, and low-grade fever. However, two subjects experienced the abrupt onset of symptoms 16 h after immunization and these reactions were consistent with serum sickness-like reactions that can occur when pre-formed antibodies react with administered antigens. One of the subjects was withdrawn, and the second received an additional immunization without recurrence of systemic AEs. Both were true-immunized subjects, precluding differentiation of the effects of mosquito saliva from the effects of sporozoites.

When true- and mock-immunized research subjects were compared overall, there were no qualitative differences in the numbers of local AEs for each subject. More systemic AEs were recorded in the true-immunized group than in the mock-immunized group, but the number of systemic AEs was small in both groups, precluding meaningful comparison. Thus, these data support the hypothesis that the reactogenicity and systemic AEs seen following immunization with PfRAS via mosquito bite result primarily from the complex mixture of mosquito antigens in the saliva [52, 53]. This is consistent with the minimal reactogenicity reported when aseptic, purified, cryopreserved sporozoites have been administered intradermally, subcutaneously or intravenously in the absence of mosquito saliva [16, 17, 54–61].

PfRAS efficacy appears to be characterized by a threshold effect greater than 1000 infectious bites induced protection in 13/14 (93 %) subjects in the early studies, while protection was only 33 % (five out of 15) in subjects immunized with fewer than 1000 infectious bites, although there were subjects protected after receiving as few as 400 irradiated infectious bites [15, 62]. A similar threshold has been identified in studies of PfSPZ vaccine [16]. In this study, only five/ten (50 %) subjects, all of whom received at least 1000 infectious bites, were protected against CHMI. This was an unexpected decrease in sterile protection from the results obtained previously [15]. Several hypotheses can be advanced to explain this difference. Firstly, the observed variability in sterile protection may be due to host genetics, biting behaviour of mosquitoes, salivary gland sporozoite counts, or other host-vector-parasite interactions. The efficacy induced by 1000 infectious PfRAS bites may range from a likely high-end estimate of >90 % to a low-end estimate of 50 % depending on these factors, particularly when the total sporozoite dose is near the protective threshold. Differences in the immunization and CHMI procedures may also have played a role, such as the longer interval between the final immunization and CHMI in these subjects compared to previous studies (Fig. 7). The efficacy induced by PfRAS may have declined beyond 3 weeks post immunization, due to a waning immune response. As a third hypothesis to explain the reduced protection seen in this study, the leukapheresis procedure used to collect PBMCs prior to CHMI in this study may have depleted the immune response.

The protection induced by PfRAS also appears to be durable. In the prior study, six protected subjects underwent additional CHMI 23–24 weeks after the last exposure to sporozoites, and five out of six were protected, indicating that once established, protection is durable. The same conclusion was reached in a study of PfSPZ Vaccine, where once a subject was protected using an adequate dose of sporozoites, protection against a second CHMI lasted at least 59 weeks [65]. In the current study, one non-protected subject underwent a second CHMI, but received additional immunizations between the first and second CHMI, and was protected after the second CHMI.

It is noteworthy that the non-protected subjects in this trial all showed a delay to parasitaemia. This delay was interpreted to indicate partial immunity—decreased liver stage parasite burden leading to the release of fewer parasites into the blood and therefore later onset of parasitemia. This inference rests on the assumption that growth rates in the blood were similar for the non-protected true-immunized research subjects and the non-immunized infectivity controls.

As previously reported [10, 63, 64], PfRAS-immunized subjects developed antibody responses to whole *P. falciparum* sporozoites and *P. falciparum* CSP. Although antibody responses to other pre-erythrocytic antigens were low or absent, cellular assays have demonstrated the presence of T cell responses to antigens expressed in sporozoites and liver stages (PfCSP, PfTRAP, PfEXP1), and in one study to a liver-(PfLSA1) and blood-stage antigens [14]. Sera from these PfRAS-immunized subjects have been used to screen protein microarrays to identify novel antigens recognized by these subjects as potentially contributing to protective efficacy [36, 50], as well as PBMC in cell-free transcription translation strategies [51]. Recently, sera and PBMC from these subjects were used to identify and characterize a panel of 27 novel *P. falciparum* antigens that provides evidence to further evaluate these antigens as candidate vaccines [35].

While PfRAS administered by infectious mosquito bites is not being further developed as a vaccine, recently administration of radiation-attenuated (metabolically active, non-replicating), aseptic, purified, cryopreserved *P. falciparum* sporozoites by intravenous inoculation has achieved 100 % efficacy in human trials [16]. The dose required (1.35 × 10^5^ sporozoites in five doses) was consistent with >1000 PfRAS mosquito bites that elicited up to 93 % protection, and protection last for at least 59 weeks after CHMI as tested in a small number of immunized subjects [65]. Thus, partial efficacy in mice with radiation-attenuated *Plasmodium berghei* first reported in 1967 [2], has now led 50 years later, to a vaccine shown to be highly effective in clinical trials against *P. falciparum*.

## Conclusions

These studies extend the results of previous studies and show that irradiated PfSPZ administered by the bite of >1000 infected mosquitoes induce protective immunity. However, the level of protective immunity after this regimen of immunization may not be as consistently high as previously reported, and may be influenced by variability in host genetics, mosquito biting, or trial procedures, such as the use of leukapheresis or longer intervals between immunization and CHMI. Given that higher doses of sporozoites appear to induce more robust immunity, these effects may be most apparent when the total number of sporozoites administered is near the protective threshold.

## Additional files

10.1186/s12936-016-1435-y Flow diagram of subjects immunized after the first CHMI. Four subjects who received CHMI (three from 1999-2000 and one from 2001-2002) and one subject who did not receive CHMI, received immunization 7. Two of these received immunization 8, and two subjects received immunizations 8-11. One subject (number 20) received immunization 12 (completing six immunizations after the first CHMI) and then received a second CHMI, and then received one more immunization (number 13).
10.1186/s12936-016-1435-y Adverse events experienced by two subjects who received more than six immunizations.
10.1186/s12936-016-1435-y Days between leukapheresis and CHMI for protected and non-protected subjects.

## Abbreviations

RAS: radiation-attenuated sporozoites
CHMI: controlled human malaria infection
PfSPZ: *Plasmodium falciparum* sporozoite
CSP: circumsporozoite protein
PBMC: peripheral blood mononuclear cells
FDA: Food and Drug Administration
IND: investigational new drug
IRB: Institutional Review Board
NMRC: Navy Medical Research Center
WRAIR: Walter Reed Army Institute of Research
OHRP: Office of Human Research Protections
ORA: Office of Research Administration
HSPP: human subjects protections program
AE: adverse event
ADL: activities of daily living
CBC: complete blood count
FA: immunofluorescence antibody assay
BSA: bovine serum albumin
FITC: fluoroscein isothiocyanate
IgG: immunoglobulin G
ELISA: enzyme-linked immunosorbent assay
SSP2/TRAP: thrombospondin-related adhesion protein
EXP1: export protein-1
LSA1: liver stage antigen-1
AST: apartate aminotransferase
ALT: alanine aminotransferase
HGB: haemoglobin
HCT: haematocrit test
WBC: white blood cell
RBC: red blood cell
