# Supplementary figures and images for: Mosquito bite immunization with radiation-attenuated Plasmodium falciparum sporozoites: safety, tolerability, protective efficacy and humoral immunogenicity
Source: Malar J. 2016 Jul 22;15:377. doi: 10.1186/s12936-016-1435-y (PMC4957371; doi:10.1186/s12936-016-1435-y)

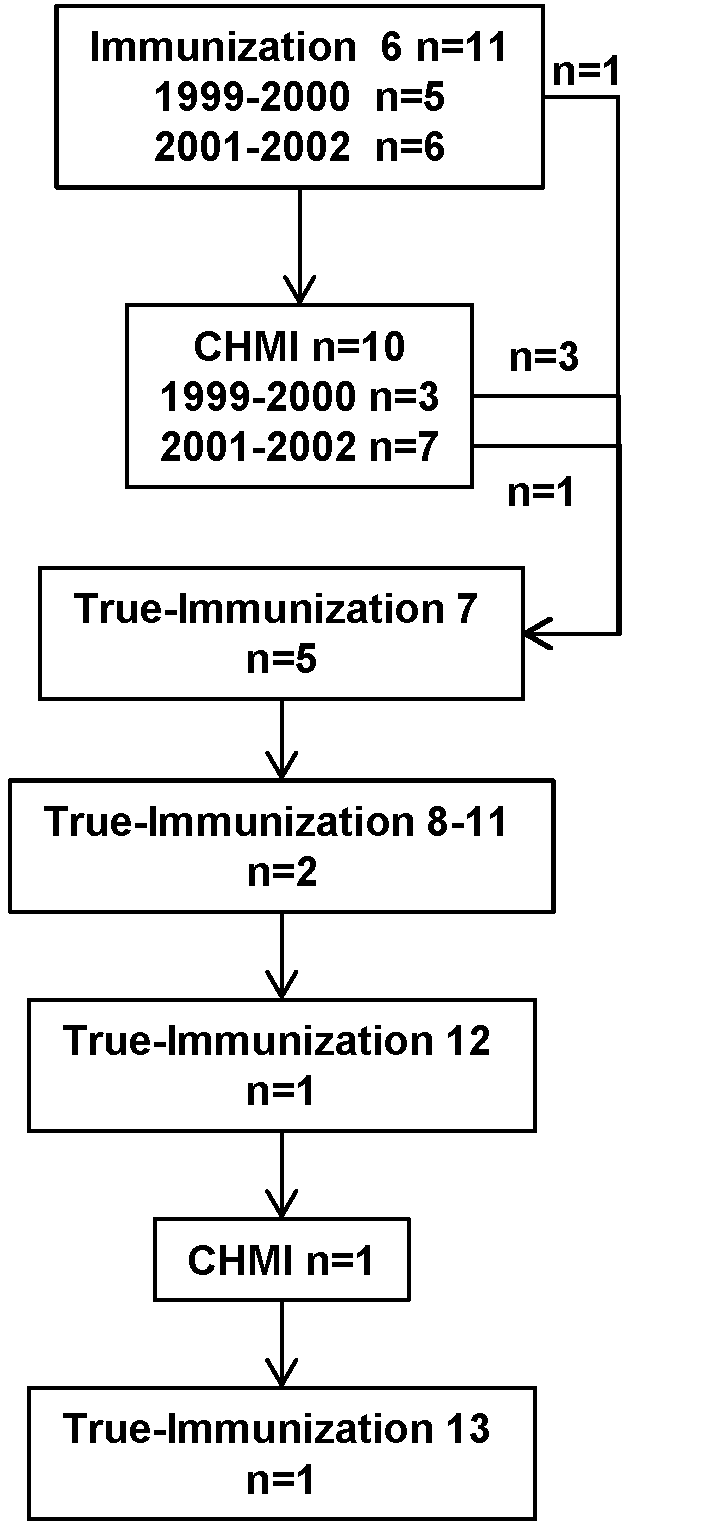

Supplement: Supplementary file 1 — 10.1186/s12936-016-1435-y Flow diagram of subjects immunized after the first CHMI. Four subjects who received CHMI (three from 1999-2000 and one from 2001-2002) and one subject who did not receive CHMI, received immunization 7. Two of these received immunization 8, and two subjects received immunizations 8-11. One subject (number 20) received immunization 12 (completing six immunizations after the first CHMI) and then received a second CHMI, and then received one more immunization (number 13). [file 12936_2016_1435_MOESM1_ESM.tiff]

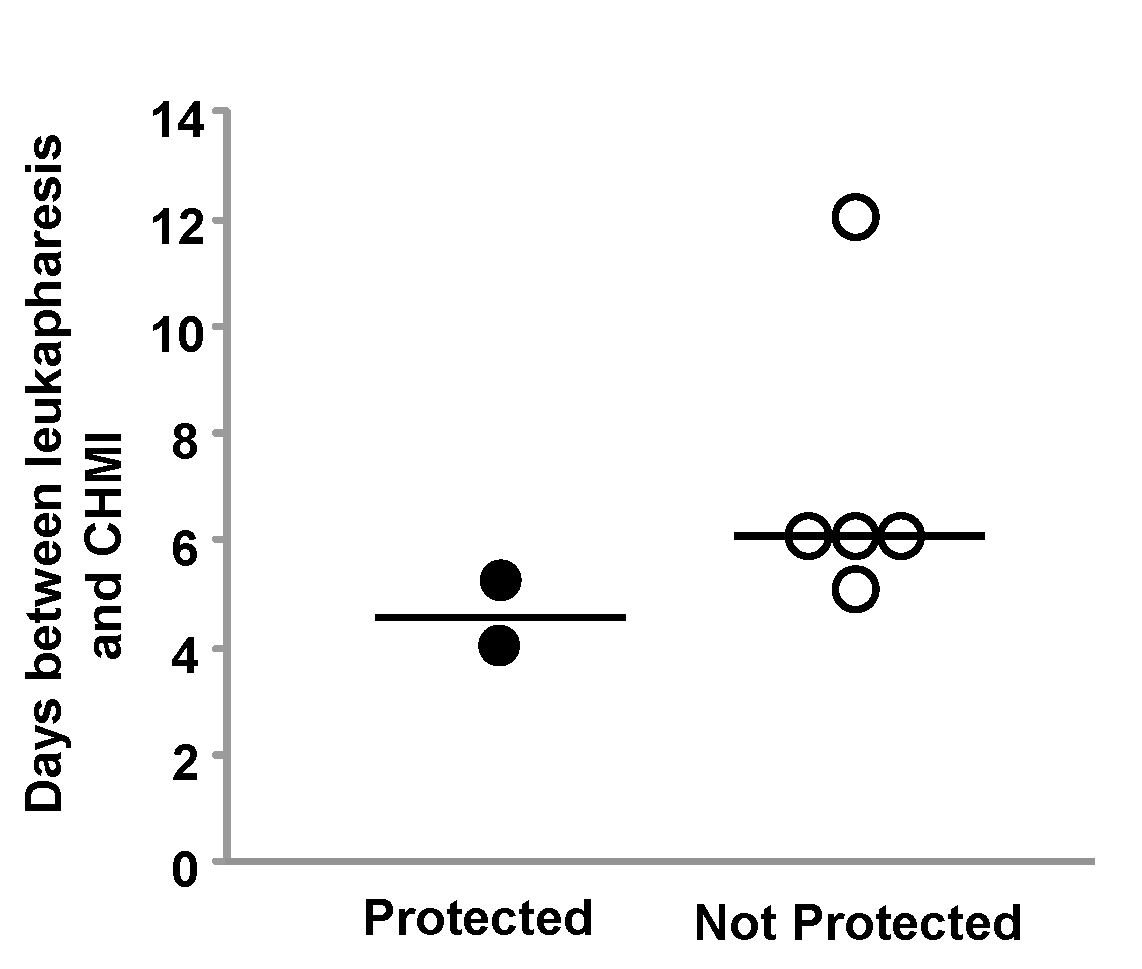

Supplement: Supplementary file 3 — 10.1186/s12936-016-1435-y Days between leukapheresis and CHMI for protected and non-protected subjects. [file 12936_2016_1435_MOESM3_ESM.tiff]
