# Supplementary material for: Mosquito bite immunization with radiation-attenuated Plasmodium falciparum sporozoites: safety, tolerability, protective efficacy and humoral immunogenicity
Source: Malar J. 2016 Jul 22;15:377. doi: 10.1186/s12936-016-1435-y (PMC4957371; doi:10.1186/s12936-016-1435-y)
Supplement: Supplementary file 2 — 10.1186/s12936-016-1435-y Adverse events experienced by two subjects who received more than six immunizations. [file 12936_2016_1435_MOESM2_ESM.docx]

**Additional file 2. Adverse events experienced by two subjects who received more than six immunizations**

Subjects number 20 and number 66 participated in further immunizations after CHMI, and both reported systemic AEs within 24 hours after immunization and are reported in the supplementary text as S1 Text.

Subject number 20 was a healthy adult male subject enrolled in 1999-2000 who had previously received six true-immunizations and CHMI (Figure 2); he then received an additional six true-immunizations and was protected to a second CHMI; he received an additional immunization #13 after the second CHMI (Figure S1). This subject had no significant past medical history but described a history of allergy to cats. He experienced AEs after the 11^th^ and 13^th^ immunizations. Three days after the 11^th^ immunization, he reported onset of fever (maximum oral temperature of 100.5 °F/38.1 °C) and malaise, the latter persisting for a further five days; he also complained of headache at 4 days and night sweats at 7 days after immunization. A malaria blood smear at 7 days was negative. His symptoms had all resolved by 8 days. There were no systemic symptoms associated with the immunization #12. After the 13^th^ immunization, he notified a study investigator at 6:00 a.m. that he had awoken at 2:00 a.m. (16 hours after immunization) with profuse (drenching) night sweats, a maximum oral temperature of 103°F (39.4°C) and shaking chills and had self-medicated with 400 mg of ibuprofen at 4:00 a.m. During the 6:00 a.m. call, he complained of fatigue, mild headache, and slight neck pain but no neck stiffness and no longer had shaking chills. The subject agreed to increase his fluid intake and at 1:00 p.m. was seen by a study investigator when he continued to complain of fatigue, malaise, and mild headache; however, his physical exam was normal. Over the next 24 hours, all of his symptoms resolved. A complete blood count, a chemistry panel, liver function tests, renal function tests, and urinalysis from the 1:00 PM visit were all normal except for the white blood cell differential count, which showed increased polymorphonuclear (PMN) cell and monocyte counts and a decreased lymphocyte count (72% PMNs [upper limit of normal 67%], 16% lymphocytes [lower limit of normal 21%], 11% monocytes [upper limit of normal 8%]), with the total WBC 6.8 cells/mm^3^ (normal 4-11). A thick blood smear was negative for malaria. IgA, IgE, IgG, and IgM levels were all normal. This subject completed the study.

Subject number 66 was a healthy adult male enrolled in 2001-2002 with medical history that was significant for inflammatory bowel syndrome (quiescent), hypertriglyceridemia, spondylosis (L5/S1), and seasonal allergies. He had received five true-immunizations and was protected to CHMI (Figure 2). After CHMI, he received his sixth immunization on the same day as subject #20 received his 13^th^ immunization, using the same batch of infected mosquitoes. The next day, the subject notified a study investigator that he had awoken at 2:00 a.m. (16 hours post immunization) with mild myalgia, maximum aural temperature of 99.8 F (37.7 °C) and that he felt “disconcerted”. Prior to leaving for work, he noted myalgia (back and shoulders), nausea and an aural temperature of 99.4 °F (37.4 °C) and self-medicated with Tylenol 650 mg, Zantac 150 mg, and one tablet (unknown dose) of Allegra at 6:30 a.m. On his way to work, he had an automobile accident in which he was rear-ended during rainy conditions. He was seen in the Emergency Department of National Naval Medical Center where he received 800 mg of ibuprofen and had a collar placed on his neck. At 2:30 p.m., he was seen by a study investigator. The subject stated that he felt normal except for fatigue and bilateral neck pain (paraspinal and trapezius areas) which he attributed to the car accident. The physical exam was normal except for mild tachycardia of 98 beats per minute. Blood was drawn for laboratory analysis and the subject was released to return to work. During a follow-up phone call the next day, the subject noted resolution of his symptoms except for residual neck pain when he removed the collar. From the blood drawn a day after his sixth immunization, a complete blood count, chemistry panel, liver function tests, renal function tests, and urinalysis were normal except for the white blood cell differential which showed increased PMN cell count and decreased lymphocyte count (74% PMNs [upper limit of normal 67%] and 13% lymphocytes [lower limit of normal 21%]), with a total WBC of 6.0 cells/mm^3^ (normal 4-11). IgA, IgG, and IgM levels were normal. The malaria blood smear was negative. This subject was not formally withdrawn and completed the study.

In reviewing the subject’s chart, he had less severe, but similar AEs, one day after his immunization number 2, when the subject complained of malaise, muscle aches and nausea. He had not taken his temperature and denied chills or sweats. At the time, the subject had recently been exposed to someone with a viral syndrome. The assessment made at the time by the study investigator was that the subject likely had a viral syndrome. In retrospect, this may have been related to immunization. No systemic symptoms were noted for the intervening immunizations (number 3, number 4, and number 5).
